# Generalization gradients for fear and disgust in human associative learning

**DOI:** 10.1038/s41598-021-93544-7

**Published:** 2021-07-09

**Authors:** Jinxia Wang, Xiaoying Sun, Jiachen Lu, HaoRan Dou, Yi Lei

**Affiliations:** 1 Institute for Brain and Psychological Sciences, Sichuan Normal University, Chengdu, 610066 China; 2 Faculty of Education and Psychology, University of Jyvaskyla, Jyväskylä, Finland; 3 Ningxia College of Construction, Ningxia, 750021 China; 4 School of Psychology, South China Normal University, Guangzhou, 510631 China; 5 Center for Neurogenetics, Shenzhen Institute of Neuroscience, Shenzhen, 518057 China

**Keywords:** Human behaviour, Trauma

## Abstract

Previous research indicates that excessive fear is a critical feature in anxiety disorders; however, recent studies suggest that disgust may also contribute to the etiology and maintenance of some anxiety disorders. It remains unclear if differences exist between these two threat-related emotions in conditioning and generalization. Evaluating different patterns of fear and disgust learning would facilitate a deeper understanding of how anxiety disorders develop. In this study, 32 college students completed threat conditioning tasks, including conditioned stimuli paired with frightening or disgusting images. Fear and disgust were divided into two randomly ordered blocks to examine differences by recording subjective US expectancy ratings and eye movements in the conditioning and generalization process. During conditioning, differing US expectancy ratings (fear vs. disgust) were found only on CS-, which may demonstrated that fear is associated with inferior discrimination learning. During the generalization test, participants exhibited greater US expectancy ratings to fear-related GS1 (generalized stimulus) and GS2 relative to disgust GS1 and GS2. Fear led to longer reaction times than disgust in both phases, and the pupil size and fixation duration for fear stimuli were larger than for disgust stimuli, suggesting that disgust generalization has a steeper gradient than fear generalization. These findings provide preliminary evidence for differences between fear- and disgust-related stimuli in conditioning and generalization, and suggest insights into treatment for anxiety and other fear- or disgust-related disorders.

## Introduction

Generalization of fear is the transfer of a conditioned response (CR) to other similar but safe stimuli that resemble the original conditioned stimulus (CS)^1–3^. Overgeneralization of fear can be maladaptive, leading individuals to excessive avoidance of safe stimuli and potentially contributing to certain anxiety disorders, such as post-traumatic stress disorder and panic disorder^4–6^. Given the important role fear generalization plays in psychological trauma, it is necessary to further elucidate this relationship to optimize anxiety-related treatment^7,8^.

The classical paradigms of research on anxiety disorders are mainly based on Pavlovian conditioning^9^. In the classical fear generalization paradigm, a neutral CS (e.g., a 500-Hz tone) is initially paired with an aversive unconditioned stimulus (US; e.g., an electric shock); over time, presenting the CS alone or a generalized stimulus (GS; e.g., a 600-Hz tone) can also come to elicit the CR (e.g., increased heart rate)^10,11^. In most previous studies of fear generalization, mild electric shocks^12^, pictures of snakes or spiders^13^, or loud screams^4^ have been used as traditional aversive US to induce a fear response.

The existing problem in such paradigms is that the aversive stimulus used as the US can often evoke both fear and disgust. Rádlová et al.^14^ demonstrated that snakes are perceived as either frightening or disgusting depending on their characteristics, including color, body size, and texture. Further, unpleasant sounds, like metal scraping over a piece of slate, are alternatives to traditional US used with children and adolescents^15^. Some people describe this sound as a “chill-sending screech” that is akin to torture. Notably, however, very few studies have addressed the confusion between fear and disgust in the conditioning and generalization processes. Some researchers assert that they have used “threatening” or “fear-evoking” stimuli as US; however, the stimulus materials (e.g., International Affective Picture System—IAPS)^16^ are, in fact, “negative,” but not necessarily threatening or fear-evoking^17^. Many aversive IAPS images (including images “causing strong dislike or disinclination”) representative of salient threats (e.g., images of injuries, mutilations, or burn victims) elicit stronger disgust responses than fear responses^18^.

Fear conditioning provides a cognitive-behavioral model of obsessive–compulsive disorder (OCD), the central symptom of which is fear of contamination^19–21^. However, the Pavlovian fear conditioning model may not fully reveal the development and maintenance of OCD symptoms. Increasing evidence has shown that disgust is the predominant emotion in OCD^22^ and is associated with repetitive, irrational behavior, such as compulsively washing one’s hands or cleaning. For example, after taking out the trash, an individual with OCD may feel unclean even after excessive handwashing. This shows how Pavlovian disgust conditioning works, in which a neutral stimulus (hand) becomes an object of disgust after it is paired with the US (trash). Most Pavlovian conditioning research has focused on fear-conditioning, generalization, and extinction, while associative learning studies on disgust are limited. Thus, gaining a better understanding of disgust conditioning in contrast to fear conditioning has clinical relevance.

We approached the current study from the perspective that fear and disgust are two independent emotions^23,24^. They differ in the display of facial expressions, behavioral responses, physiological responses, and the mechanisms of action and brain activity. Nevertheless, the similarities between fear and disgust make it difficult to disentangle one from the other in terms of emotion elicitation^25^. Specifically, both are unpleasant emotions associated with threat and are often involved in clinical disorders, such as (OCD) and agoraphobia. A disabling and irrational fear of animals is a common phobia, with an incidence rate of 3.3–5.7%^26^. Typically, snakes have been found to trigger anxiety in 53.3% of the population, and spiders have also been shown to elicit unreasonable fear in many people^27^. Previous studies have shown that the fear of animals can be divided into two categories: predatory animals (e.g., tiger, bear) and fear-relevant animals (e.g., rat, spider)^28^. Fear of animals in the fear-relevant category was found to be mediated by individual levels of disgust sensitivity; however, no correlation was found between disgust sensitivity and the fear of predatory animals^29,30^. These findings suggest a disease-avoidance model of animal-related fears, and that such phobias are evolutionarily mediated by the disgust response^31,32^. This accounts for a considerable number of studies targeting negative emotions in general instead of specific emotions.

Klucken et al.^24^ investigated the neural network underlying fear- and disgust-conditioned responses using functional magnetic resonance imaging, and revealed that both aversive CRs shared the same ROI-activations, including the cingulate cortex, nucleus accumbens, orbitofrontal cortex, and occipital cortex. In addition, insular activation was found to be sensitive to disgust conditions. Further, compared with fear-associative CS+, disgust-CS+ paired with disgust stimuli will elicit attentional avoidance^33^. For example, individuals with blood-injection-injury phobia respond with elevated disgust rather than fear to threat-based US (e.g., blood, injections, and bodily mutilations), suggesting that disgust, but not fear, plays a vital role in the development of blood-injection-injury phobia^34^. Therefore, it is important to elucidate how individuals differ in their responses to fear- and disgust-related associative learning.

While fear targets impending danger that may cause physical harm^35^, disgust motivates individuals to protect themselves from contamination or disease, thus targeting revolting threats that are usually less urgent^36^. Since disgust serves as a contamination-avoidance system, experiencing disgust is accompanied by avoidance and suppressed sensory acquisition^37^. Whereas fear increases visual input and leads to increased attention, disgust shows the opposite pattern. Based on previous studies, we hypothesized a greater US expectation for CS+ than for CS- in both fear and disgust conditioning phases, and a longer reaction time for fear-related CS than for disgust-related CS. During the generalization test, we assumed that fear-related GS1 and GS2 would evoke larger US expectations than would disgust-related GS1 and GS2. Further, we hypothesized pupil enlargement would be greater for fear-related CS+, GS1, and GS2 than for disgust-related stimuli.

## Methods

### Participants

In the current study, we recruited 32 respondents (18 women; age range: 18–23; mean age = 21.22 years, SD = 1.47) among undergraduate students from Shenzhen, China, who each received approximately 70 RMB for their participation. An a priori calculation of statistical power (G*Power) suggested that the recruitment target should be 28 participants to achieve a medium effect size of 0.20, an alpha level of 0.05, and a 1-beta level of 0.80^38,39^. Thus, the recruited sample (*N* = 32) was large enough to detect an effect at the significance level of α = 0.05. All participants met the following criteria: right-handed, normal or corrected-to-normal eyesight, no previous traumatic experiences, and no neurological diseases or drug abuse. The participants were informed that they could quit the experiment at any time and were asked to sign an informed consent form before the experiment began. The experimental protocol was established, according to the ethical guidelines of the Helsinki Declaration and was approved the Ethical Committee of Shenzhen University.

## Materials

### Unconditioned stimulus

We asked 115 participants (54 women; mean age = 21.92 years, SD = 1.43) from China to provide as many fear-inducing (e.g., snake) or disgust-inducing (e.g., cockroach) nouns as possible through a free-association task. We then selected the 180 most frequent stimuli (each category contained 90 different pictures) and classified those into three categories—animals, scenes, and objects—with 30 images per category. The 180 realistic photographs were obtained from 3 web sources: Baidu, Souhu, Sogou. All pictures were in the public domain and not permit for commercial use. We used verbal description to illustrate the materials. Examples of fear images: (1) Animals: tiger, lion, snake, shark; (2) Objects: knife, gun, ghost face, skeleton; (3) Scenes: high places, enclosed spaces, operating room. Examples of disgust images: (1) Animals: maggot, fly, cockroach; (2) Objects: knife, gun, ghost face, skeleton; (3) Scenes: open dump, sewage, toilet. Next, we enrolled 84 participants (39 men, mean age = 20.55 years, SD = 1.43) to assess disgust, fear, valence, and arousal ratings for each stimulus on a nine-point scale. Finally, a total of 81 fear-evoking and 84 disgust-evoking pictures were chosen. Independent *t*-tests revealed that the mean fear ratings for the fear-evoking category (*M* = 4.80; *SD* = 1.06) were significantly higher than for the disgusting-evoking category (*M* = 3.32; *SD* = 0.86; *t*(80) = 12.715, *p* < 0.001, Cohen’s d = 0.87), and the mean disgust ratings for the disgust-evoking category (*M* = 5.84; *SD* = 1.21) were significantly higher than for the fear-evoking category (*M* = 4.05; *SD* = 0.97; *t*(83) = 22.737, *p* < 0.001, Cohen’s d = 2.40). There was no significant difference in valence between the two categories (*t*(80) = 3.701, *p* = 0.24, Cohen’s d = 0.58), whereas the arousal from the images in the fear-evoking category (*M* = 7.62; *SD* = 6.16) was significantly higher than in the disgust-evoking category (*M* = 6.85; *SD* = 5.54, *t*(80) = 7.329, *p* < 0.001, Cohen’s d = 1.17). In the current research, we selected 30 stimuli to represent the categories of Disgusting US and Fearful US (15 each). All US stimuli in this study were selected from the 165 emotional pictures.

### Conditioned stimulus and generalized stimulus

**Figure 1:**
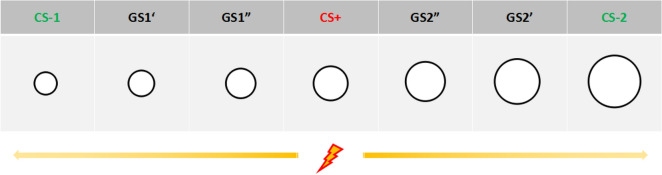
Experimental material: CS (conditioned stimuli).

The stimuli were seven black rings, continuously increasing in size (7.37–11.94 cm in diameter, 15% increments; Fig. 1)^40^. The mid-sized ring (9.56 cm) served as CS+ paired with the US (75% reinforcement), while the largest (11.94 cm, CS-1) and smallest (7.37 cm, CS-2) rings were used as CS- presented alone^41^. The remaining four rings (8.13 cm, 8.89 cm, 10.41 cm, and 11.18 cm) were used as the GS.

## Experimental procedure

The fear and disgust learning (conditioning, generalization) took place in two separate sessions, and the order was counterbalanced.

### Habituation

**Figure 2:**
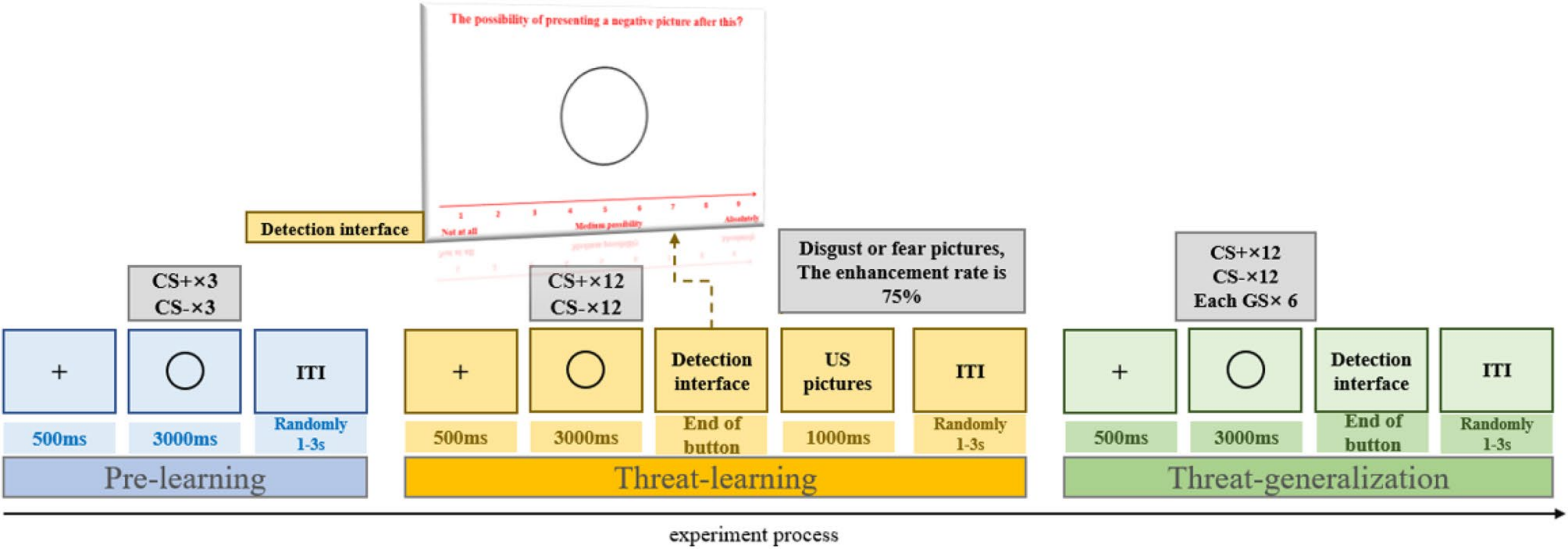
The experimental procedure.

Each CS was presented without US for 3000 ms (three times each), and no CS was repeated more than twice in a row, with a jittered inter-trial interval (ITI) ranging from 1 to 3 s (Fig. 2).

### Conditioning

The conditioning phase consisted of 12 CS+, 12 CS-1, and 12 CS-2, divided into three blocks (3000 ms duration; 36 total trials). The CS+ was partially followed by the US (1000 ms duration; 75% reinforcement rate), and the CS- (i.e., CS-1; CS-2) was presented alone. The ITI was jittered between 1 and 3 s. During each trial, participants were asked to rate the level of US expectancy on a nine-point scale (1 = *least likely*, 5 = *moderately likely*, 9 = *most likely*), and eye movement responses were recorded.

### Generalization

The generalization phase included six blocks with eight trials in each block: both the CS+ and CS- were presented twice and each of the four GS was presented once (3000 ms duration). To avoid extinction, all the CS+ in the generalization phase were paired with a US. For each trial, participants were asked to assess the US expectancy via a nine-point rating scale, and the ITI ranged between 1 and 3 s.

### Acquisition and analysis of eye movement indicators

Eye movement tracking was recorded with the Eye-Link 1000 desktop eye movement recorder (sampling rate: 1000 Hz). We focused primarily on participants’ eye responses during the first exposure to threat stimuli (US) in the acquisition phase. The main eye movement variables changed in pupil diameter and initial gaze time when the CS appeared.

### Statistical analysis

Analyses were performed with SPSS version 20.0 for IBM. For the conditioning phase, a 3 (stimulus type: CS+, CS-1, CS-2) × 2 (emotion type: fear, disgust) repeated-measures ANOVA was conducted on the subjective expectation scores and reaction times. The data for pupil size and fixation duration were analyzed using one-way ANOVAs with emotion (fear/disgust/neutral) as an independent variable. For the generalization phase, a 5 (stimulus type: CS+, CS-1, GS-1, CS-2, GS-2) × 2 (emotion type: fear, disgust) repeated-measures ANOVA was performed on the US expectation scores and reaction times (the statistical significance level was *p* < 0.05).

## Results

### Conditioning phase

#### Subjective expectation score

A repeated-measures ANOVA demonstrated that the main effect of emotion type was significant (*F*(1,31) = 3.46, *p* = 0.021, *η*_*p*_^2^ = 0.08), and the expectation of fear was greater than that of disgust (Table 1). The main effect of stimulus type was significant (*F*(2,62) = 15.69, *p* < 0.001, *η*_*p*_^2^ = 0.35), and the expectation of CS+ was greater than that of CS-1 and CS-2. The interaction between emotion type and stimulus type was significant (*F*(2,62) = 15.69, *p* < 0.001, *η*_*p*_^2^ = 0.35). Bonferroni corrected post hoc analysis revealed that the subjective expectation scores of CS-1 (2.59 ± 0.92) and CS-2 (2.74 ± 1.17) under the fear condition were higher than those of CS-1 (1.63 ± 0.38) and CS-2 (1.67 ± 0.46) under the aversion condition. However, the fear-CS + (5.95 ± 0.66) and disgust-CS + (6.15 ± 0.72) scores were not significant (*p* > 0.05).

**Table 1:** The behavior results (M ± SD).

	Fear condition		Disgust condition	
	Subjective expectations (1–9)	Reaction time (ms)	Subjective expectations (1–9)	Reaction time (ms)
**Acquisition**				
CS-1	2.59 ± .92	2275 ± 160	1.63 ± .38	1669 ± 81
CS +	5.95 ± .66	2303 ± 108	6.15 ± .72	2006 ± 94
CS-2	2.74 ± 1.17	2379 ± 87	1.67 ± .46	1681 ± 92
**Generalization**				
CS-1	1.94 ± .16	1613 ± 157	1.59 ± .19	1136 ± 148
GS1	3.92 ± .13	2236 ± 91	3.24 ± .11	1472 ± 55
CS +	5.36 ± .17	2417 ± 134	6.25 ± .14	1513 ± 140
GS2	3.82 ± .10	2266 ± 168	2.82 ± .11	1592 ± 137
CS-2	1.92 ± .13	1782 ± 69	1.56 ± .12	1540 ± 61

#### Reaction time

There was a significant main effect of emotion type (*F*(1,31) = 51.19, *p* < 0.001, *η*_*p*_^2^ = 0.13) but not of stimulus type (*F*(2,62) = 1.32, *p* > 0.05). There was a significant interaction between emotion type and stimulus type (*F*(2,62) = 14.58, *p* < 0.001, *η*_*p*_^2^ = 0.67). Bonferroni corrected post hoc analysis indicated that the reaction times for fear-related CS-1 (2275 ± 160 ms), CS-2 (2379 ± 87 ms), and CS + (2303 ± 108 ms) were greater than those for disgust-related CS-1 (1669 ± 81 ms), CS-2 (1681 ± 92 ms), and CS + (2006 ± 94 ms).

### Generalization phase

#### Subjective expectation score

**Figure 3:**
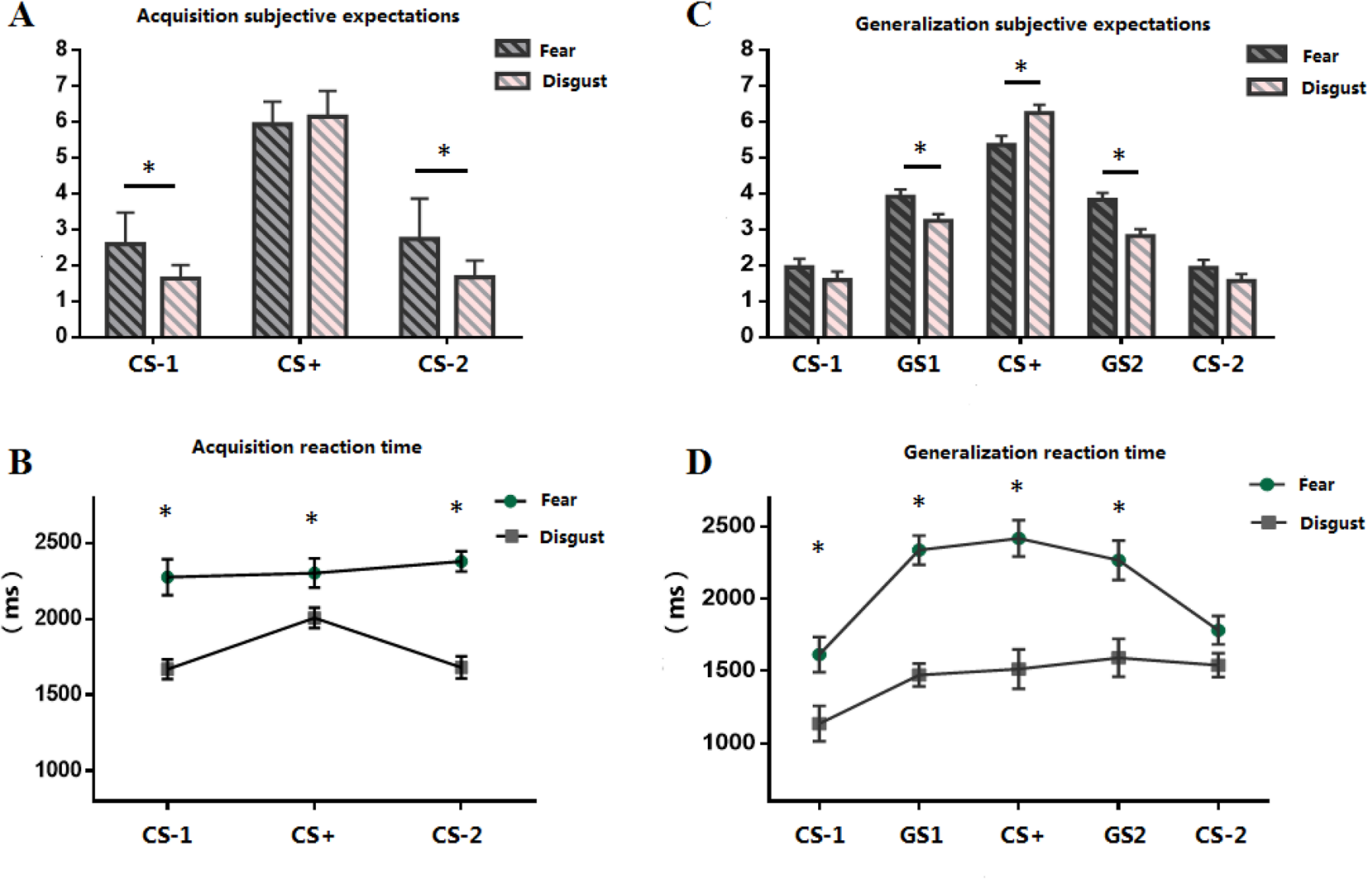
US expectancy ratings and mean response times were collected for each trial in the acquisition and generalization tasks. (**A**) greater subjective ratings for fear-CS- vs. disgust-CS-; (**B**) greater reaction times for fearbased CS compared to disgust-based CS; (**C**) US expectancy ratings of fear-related GS1 and GS2 were significantly greater than those of disgust GS1 and GS2; (**D**) shorter reaction times for disgusting GSs than to fearful GSs. *p < 0.05. Error bars represent standard mean errors. CS = conditioned stimulus; GS = generalized stimulus.

We combined the four GS into two variables, where GS1 was the average value of GS1’ and GS1,” and GS2 was the average value of GS2’ and GS2” (Fig. 3). There was a significant main effect of emotion type (*F*(1,31) = 71.79, *p* < 0.001, *η*_*p*_^2^ = 0.20), and the subjective expectation of fear-related CS was greater than that of disgust-related CS. There was a significant main effect of stimulus type (*F*(4,124) = 2.24, *p* = 0.042, *η*_*p*_^2^ = 0.06) for the subjective expectation of CS + , and GS was significantly greater than CS-1 and CS-2. The interaction between emotion type and stimulus type was significant (*F*(4,124) = 11.73, *p* < 0.001, ηp^2^ = 0.49). Post hoc analysis revealed that the subjective expectation scores for GS1 (3.92 ± 0.13) and GS2 (3.82 ± 0.10) under the fear condition were greater than those of GS1 (3.24 ± 0.11) and GS2 (2.82 ± 0.11) under the disgust condition (*F*(2,62) = 7.93, *p* < 0.001, *η*_*p*_^2^ = 0.28). The expectancy of fear-CS + (5.36 ± 0.17) was significantly lower than that of disgust-CS + (6.25 ± 0.14) (*F*(2,62) = 6.67, *p* < 0.001, *η*_*p*_^2^ = 0.54). Further, the expectation score did not vary between fear-CS-1 (1.94 ± 0.16) and CS-2 (1.92 ± 0.13) and disgust-CS-1 (1.59 ± 0.19) and CS-2 (1.56 ± 0.12; *p* > 0.05).

#### Reaction time

There was a significant main effect of stimulus type (*F*(4,124) = 5.94, *p* < 0.001, *η*_*p*_^2^ = 0.39); however, the main effect of emotion type marginally failed to reach significance (*F*(1,31) = 3.07, *p* = 0.054, *η*_*p*_^2^ = 0.06). The interaction between emotion type and stimulus type was significant (*F*(4,124) = 21.72, *p* < 0.001, *η*_*p*_^2^ = 0.81). Post hoc analysis indicated that the reaction times for fear-related GS1 (2236 ± 91 ms), CS + (2417 ± 134 ms), GS2 (2266 ± 168 ms), and CS-1 (1613 ± 157 ms) were greater than those for disgust-related GS1 (1472 ± 55 ms), CS + (1513 ± 140 ms), GS2 (1592 ± 137 ms), and CS-1 (1136 ± 148 ms); however, the reaction times between fear-related CS-2 (1782 ± 69 ms) and disgust-related CS-2 (1540 ± 61 ms) did not vary significantly (*p* > 0.05).

### Eye movement results

**Figure 4:**
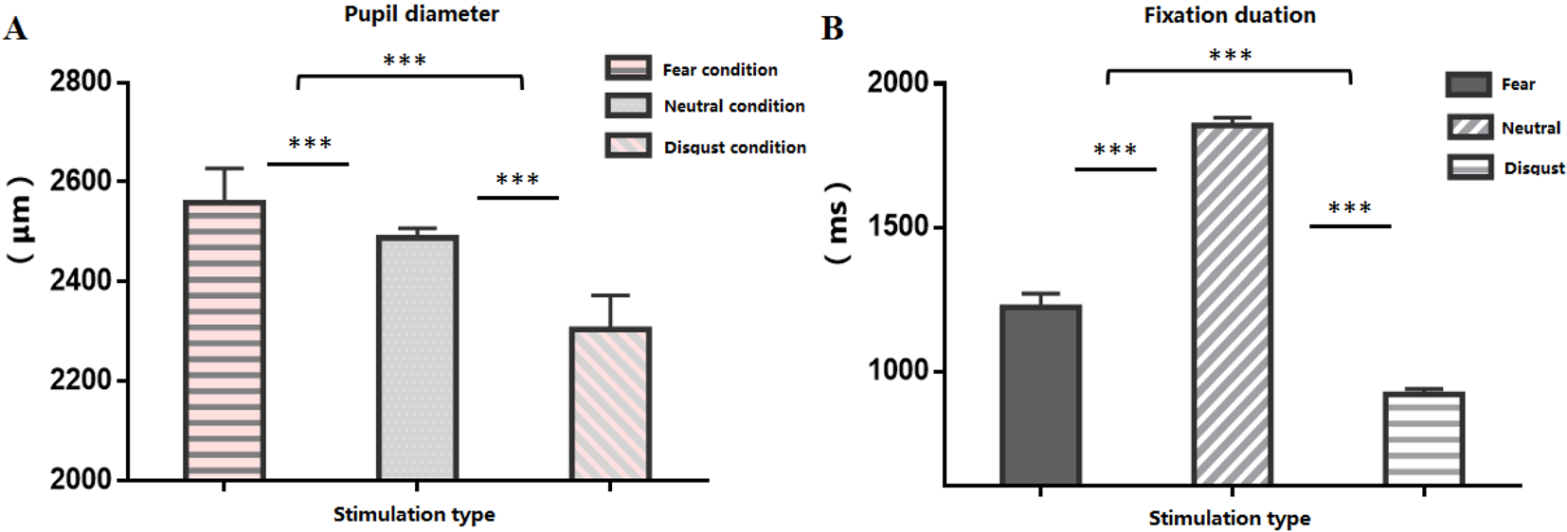
(**A**) The differences in pupil sizes between fear and disgust. (**B**) The fixation duration differences between fear and disgust. ***p < 0.001. Error bars represent standard mean errors.

The eye movement results revealed that fear- and disgust-related stimuli elicited different pupil sizes. Pupil diameter was larger for fearful stimuli (one-way ANOVA) than for disgusting stimuli (Fig. 4). When exposed to the image display, the mean pupil size of the participants under the neutral, disgust, and fear conditions was 2487.4, 2304.1, and 2558.5 μm, respectively. Post hoc comparisons indicated that the difference between any two types of emotional stimuli reached statistical significance (*p* < 0.001). There was a main effect of fixation duration in the first-time fixation. Among the stimuli, there was a significant difference between the fear and disgust conditions (*M* = 396.63, *p* < 0.001) and between the fear and neutral conditions (*M* = 572.25, *p* < 0.001). Fixation duration in disgust conditions was significantly lower than that in neutral conditions (*M* = 967.03, *p* < 0.001).

## Discussion

Most previous generalization studies have focused on fear; however, only a few studies have addressed the importance of disgust generalization, an equally influential negative emotion^42,43^. Further, the US used in many fear generalization experiments actually elicits both fear and disgust (e.g., metal scraping). To the best of our knowledge, the present study was the first to compare the generalization of conditioned fear and disgust.

In selecting the experimental US, we developed a new Threat Picture System for future research on fear and disgust by using a free-association task. The fear-eliciting images were separated from the disgust eliciting ones, and there were significant differences in the disgust, fear, and arousal ratings assessed by the participants. These two types of threat-response emotions were identical in valance but differed in arousal—frightening pictures elicited higher arousal ratings than did disgusting pictures. Moreover, the three image categories, which included animals, scenes, and objects, were compared in terms of how well they induced the various emotions. A significant effect of category on fear was observed: people rated the animal images as more frightening than the images of scenes. There was also a significant effect of category on disgust: the images of scenes induced more disgust than the animal and object images. Notably, the three image categories may be useful for different types of anxiety disorders. For example, the animal category is more suitable for specific phobias; whereas, pictures of scenes may be a better choice for research on claustrophobia or panic disorder.

Our results revealed that for fear conditioning, there was no significant difference in the subjective expectations of fear- and disgust-relevant CS + , while participants reported higher expectations for fear-relevant CS-1 and CS-2 relative to disgust-relevant CS-1 and CS-2. One explanation for this observation may be that the strength of the CS-US association in the two threat-response emotions was equally strong; however, compared to disgust, fear is associated with inferior discrimination learning. As for fear generalization, some previous studies indicated that, in general, as the difference in physical properties between GS and CS + increases, the fear response to GS decreases^40,43^. Thus, the more similar the stimulus, the more easily fear will be generalized in response. The results of the current study also supported this conclusion. In the generalization phase, the participants exhibited generalized fear or disgust toward GS which was close to CS + , while the subjective expectations of CS-1 and CS-2 remained at a consistently low level. Further, as the circle decreased in size, the learned fear almost completely disappeared. The subjective expectation scores of fear-related GS1 and GS2 were significantly greater than those of disgust GS1 and GS2, which demonstrates that fear can be generalized more broadly, relative to disgust. Taken together, disgust-eliciting GS revealed a steep generalization gradient, while fear-eliciting GS displaying a flattened gradient.

Remarkably, the CS + expectancy score for disgust was significantly higher than the disgust-CS + score for fear, even though they were equal in the conditioning phase. This might suggest that disgust is more resistant to extinction than fear, which is similar to previous findings^44–46^ demonstrating that recall and recognition are greater for disgusting stimuli relative to either frightening or neutral stimuli. In sum, abundant research indicates that extinction treatment is less effective in reducing disgust than fear. This finding has immediate clinical relevance, as generalization and extinction are important for contamination-based OCD.

The differences in reaction times also confirmed the differences in the conditioning and generalization processes under the two emotional conditions. Participant response time to fear-based CS + was shorter than that for fear-based CS-; in contrast, the response time for disgust-based CS displayed the opposite trend. In general, the participants responded more slowly to fearful stimuli than to disgusting stimuli. Fear is more closely related to survival and can mobilize people’s attention as well as elicit a longer reaction. It is a “stop to observe”^47^ behavior pattern, and is different from disgust, which is characterized by a “just want to escape quickly” behavior pattern^48,49^. Although both emotions are threat-related, fear is thought to elicit an instinctive response to deal with an immediate threat, and is expressed as the expansion of sensory perception, attention, and feelings in response to the surrounding environment^37^. Meanwhile, disgust elicits immediate sensory rejection to avoid contamination, expressed as the contraction of the pupils’ dilation and resentment and avoidance of disgusting objects^50^. Notably, both pupil size and fixation duration in response to fear stimuli were larger than those in response to disgust stimuli. This phenomenon may suggest different evolutionary responses to these two types of threatening emotions.

The experimental procedure of the current study was well controlled, and the paradigm integrated test performance (response to stimuli) to behavioral measures (eye movements and changes in pupil size). This research markedly distinguished fear from disgust, thus contributing to the clarification of their different natures on an experimental level. Nevertheless, the present study has some limitations. First, we did not use standardized tools to verify inclusion criteria such as right-handedness, normal eyesight, and the absence of previous traumatic experiences. Future studies should use standardized tools (e.g., the Edinburgh Handedness Inventory) instead of generic questions. Second, our sample included only healthy participants; thus, our results may not be generalizable to patients with anxiety disorders. It is necessary to study patients with OCD as a special group. Finally, improving the ecological validity of fear generalization laboratory research is also a direction for future research^51^. Most empirical studies use Pavlovian conditioned reflexes as the experimental paradigm, and the generalized CR along with the perceptual similarity to CS + in simple sensory dimensions (e.g., sound, color, and shape). However, stimuli in real-life situations usually involve multiple sensory dimensions, and individuals' emotional experiences and knowledge also affect their perception of stimuli^52^. Thus, future research could examine the associations between fear, disgust, and anxiety disorders using category-based generalization.

In summary, the current study demonstrated that disgust can be more resistant to extinction relative to fear when the strength of the CS-US association is the same. In the generalization phase, the subjective expectations of disgust-GS were significantly higher than those of fear. We concluded that the fear generalization gradient is more flattening than the disgust generalization gradient. Further, fear-related GS led to longer reaction times compared to disgust-related GS, and the pupil size and fixation duration for fear stimuli were larger than those for disgust stimuli, indicating that individuals exhibited an attention bias toward fear-based CS and GS. These findings have notable clinical implications, particularly for intervention techniques related to fear and disgust and for learning mechanisms, anxiety disorder treatments, and interventions for emotional development in children.
